# Increasing importance of protein flexibility in designing biocatalytic processes^☆^

**DOI:** 10.1016/j.btre.2015.04.001

**Published:** 2015-04-02

**Authors:** Joyeeta Mukherjee, Munishwar Nath Gupta

**Affiliations:** a Department of Chemistry, Indian Institute of Technology Delhi, Hauz Khas, New Delhi 110016, India; b Department of Biochemical Engineering and Biotechnology, Indian Institute of Technology Delhi, Hauz Khas, New Delhi 110016, India

**Keywords:** Protein flexibility, Enzyme promiscuity, Intrinsically disordered proteins, Enzyme enantioselectivity, Enzymes in organic solvents

## Abstract

Enzymes require some flexibility for catalysis. Biotechnologists prefer stable enzymes but often this stabilization comes at the cost of reduced efficiency. Enzymes from thermophiles have low flexibility but poor catalytic rates. Enzymes from psychrophiles are less stable but show good catalytic rates at low temperature. In organic solvents enzymes perform poorly as the prior drying makes the enzyme molecules very rigid. Adding water or increasing reaction temperature improves flexibility and catalytic rates. In case of hydrolases, flexibility and enantioselectivity have interdependence. Understanding the complex role of protein flexibility in biocatalysis can help in designing biotechnological processes.

## 1 Introduction

It was the early workers in the area of protein structure who pointed out the importance of conformation (even before organic chemists) in defining the function of the protein molecule. So, establishing structure–function relationships in biological systems became the focus of the investigations and it continues till date (albeit new terms evolve to refer to it, the first one was molecular biology) [36]. Lindstrom Lang’s suggestion to refer to various levels of organization of the protein structure as primary, secondary, tertiary and quaternary structures created a useful framework for studying structure–function relationship [57]. The importance of conformational flexibility was highlighted by Koshland’s induced fit theory [38]. At that point in time, the inherent contradiction was not apparent between the importance of an ordered structure and yet the necessity of it being flexible. Many decades later, we are confronted by that. As much as 30% of the eukaryotic proteins are intrinsically disordered proteins (IDPs) and in fact depend upon that “lack of ordered structure” for their biological function [49]. As Mittag et al. points out, neither the lock and key mechanism presuming a rigid protein structure, nor Koshland’s induced fit mechanisms of molecular recognition explain their biological function [49]. The induced fit mechanism is limited to either few side chains or re-organization of domains. In the case of the IDPs all those descriptions do not make any sense as they presume structured protein segments; IDPs seem to derive the advantage from lack of structure during their biological function, for example, while working as “hub proteins”. Such hub proteins use “one-to-many binding modes” [73] by exploiting the structural disorder and assume the required different conformations during binding with different partners. Some authors have talked of ‘preformed elements’ or ‘molecular recognition features’ [49]. The cost for this behaviour is paid by their vulnerability to aggregation. Many degenerative diseases result from aggregation of IDPs and that includes numerous neurodegenerative diseases like Alzheimer’s disease and Parkinson’s disease [74], [41]. Considering that vulnerability to aggregation and stability have more or less one to one correlationship, it is frustrating that a very subtle change in the native structure can result in protein forming amyloid aggregates. Bemporad and Chiti have referred to “native-like structures” becoming more prone to aggregation [5]. Biotechnologists have a stake in the way our understanding of the “importance of well defined structure” versus the lack of it in biological function is changing. The present mini-review lists some key reasons for that in different contexts of applied biocatalysis.

## 2 Biotechnologists tend to favour stable proteins

Extensive efforts have been made by biotechnologists to stabilize proteins/enzymes. Chemical modification, protein immobilization, chemical crosslinking and protein engineering have all been used to enhance stability of the enzymes [51], [26], [68], [54], [72], [28], [48], [70], [22]. Most of these studies are directed towards reducing their vulnerability to high temperature but there have been efforts about enhancing stability towards other ‘stress’ conditions as well. Alkaline proteases (more active and stable at high pH) are required as detergent enzymes [69]. Enzymes more stable in organic solvents has now become a very active area of research [17], [48], [1]. Understanding stability under high pressure conditions has also been carried out [29].

While these are undoubtedly desirable studies, in the pursuit of more stability, we have often overlooked “collateral damage”. Immobilization, for example, is often accompanied by increased mass transfer constraints [15], [10]. Most of the text books while discussing the Michaelis Menten kinetics, unfortunately end up implying that decreasing *K*_m_ (better association constant for the ES complex formation) results in a more efficient enzyme. As Fersht, in his seminal book [16], discusses it fairly succinctly, nature while designing more efficient enzymes aims at improving *k*_cat_/*K*_m_. For achieving a desired *k*_cat_/*K*_m_ value, “the enzyme evolves to increase *K*_m_” [16]. Binding can be easily improved (as reflected in the low value of *K*_m_) by increasing rigidity. The whole approach of bioimprinting of proteins is based upon that [47]. Good *k*_cat_ is favoured by flexibility.

## 3 Lessons from extremophiles

Enzyme catalysis occurs *in vivo* at sub-zero temperatures to hot springs [50], [7], [12], [14], [71], [53]. The enzymes from psychrophilic organisms tend to be very flexible, have good catalytic activity bur poor thermal stability. The enzymes from thermophiles generally tend to be very stable at high temperatures; however rigid conformations result in their not being very efficient catalysts [14], [71], [53].

As early as 1993, Jaenicke [33] pointed out that “molecular adaptation obviously results in optimum protein flexibility rather than maximum stability”. It is worth noting that if metabolic rates are normalized to temperatures of the normal habitats of the microorganisms, enzymes show metabolic rates in the same range. The changes in protein–substrate interactions with temperature play a compensatory role to protein flexibility during this adaptation [30].

The studies on enzymes from psychrophiles reveal that increase in flexibility is largely achieved by altering the structural traits in the reverse direction as compared to enzymes from thermophiles [19]. “The current consensus is that only subtle modifications of the conformation of cold adapted enzymes can be related to the structural flexibility and that each enzyme adopts its own strategy. Moreover, it appears that there is a continuum in the strategy of protein adaptation to temperature, since known structural factors involved in protein stability of thermophiles are either reduced in number or modified, in order to increase flexibility in psychrophilic enzymes” [18]. Possibility of operating biocatalytic processes at low temperature (with reduced energy consumptions) makes these enzymes quite attractive choices. For continuously operated processes, microbial growth is a worrisome factor; operating such processes at low temperature with enzymes from psychrophiles minimizes that possibility. Excellent reviews on the biotechnological applications of enzymes from psychrophiles are already available [18], [12], [20].

## 4 Biocatalysis in organic media

**Fig. 1:**
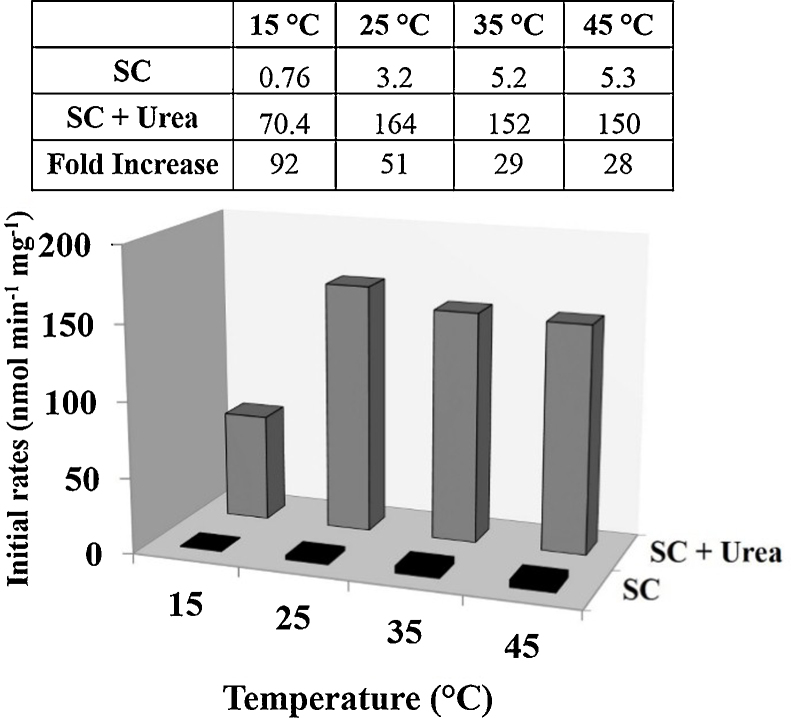
Effect of temperature on subtilisin catalysed transesterification reaction in *n*-hexane. The samples of SC and urea treated SC lyophilized for 48 h were then used for the transesterification reaction between *N*-acetyl-L-phenylalanine ethyl ester and *n*-propanol. The inlay shows the fold increase between the lyophilized SC and the SC lyophilized with urea. The reaction was carried out at various temperatures. Initial rates of transesterification were determined by estimating the aliquots taken at different time intervals by HPLC. The reactions in each case were carried out in duplicates and the results within each set agreed within 3%.

Reproduced with permission by Elsevier from Ref. [52] Tetrahedron Lett., http://dx.doi.org/10.1016/j.tetlet.2015.02.109.

The possibility of carrying out biocatalysis in media other than aqueous buffers has been found to be extremely useful for biotechnologists. Nearly anhydrous organic solvents, aqueous–organic co-solvent mixtures, water–organic solvent biphasic systems, reverse micelles and ionic liquids represent such non aqueous media [46], [24], [11], [25], [27], [55], [32], [62], [63], [13]. The ensuing discussion will mostly pertain to nearly anhydrous organic solvents. The high stability which enzymes normally have in this kind of media was “dramatically” shown by Klibanov and Zaks reporting the survival of a lipase when placed at 100 °C in 99% organic medium [76]. While this excellent result does not seem to have been utilized much by biotechnologists for carrying out biotransformations at 100 °C, the subsequent unfolding story has revealed that this high stability, originating from the very highly rigid structure (which enzymes acquire in such media) is accompanied by poor *k*_cat_/*K*_m_ as compared to the corresponding *k*_cat_/*K*_m_ values of enzymes in aqueous buffers [40]. This has led to a somewhat interesting situation that subtilisin “denatured” by 6 M urea in aqueous buffers, when dried up, actually shows very high activity in anhydrous *n*-hexane (as compared to the untreated enzyme) [23], [52] (Fig. 1).

## 5 Enantioselectivity and catalytic promiscuity

**Fig. 2:**
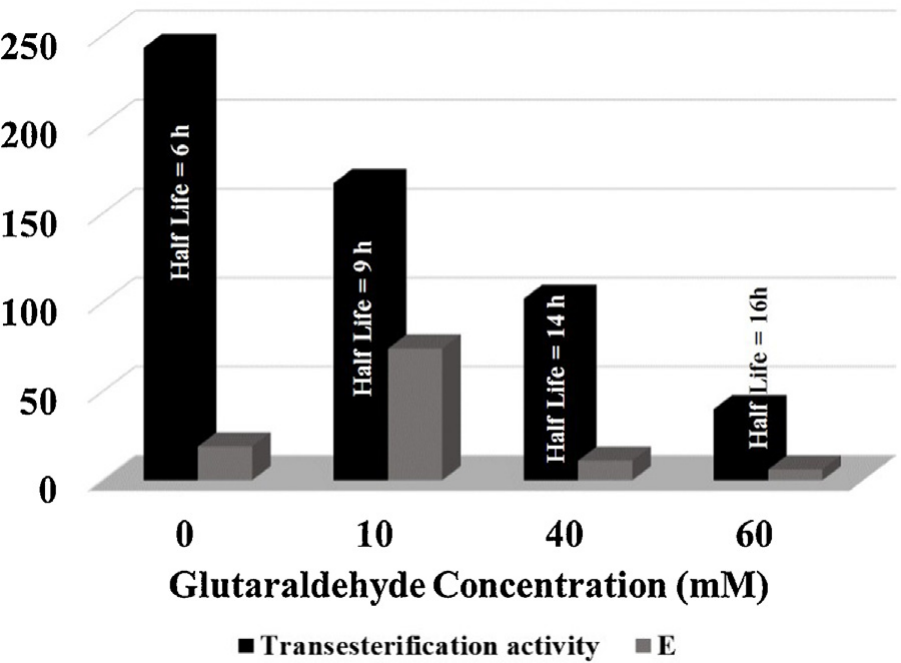
Performance of the cross-linked enzyme aggregates (CLEAs) made with various amounts of glutaraldehyde in anhydrous solvent free medium during the transacetylation of citronellol with vinyl acetate using *Burkholderia cepacia* lipase. The half lives were measured at 55 °C.

Adapted from Ref. [44] Biocatal. Biotransform., 26, 235–242.

The effect of flexibility is not just limited to enzyme stability or catalytic rates alone. It affects enantioselectivity as well. Cross-linked enzyme aggregates (CLEA) is a very well known example of carrier free immobilization [65], [64]. CLEAs perform well in both aqueous and non-aqueous media [61], [64], [44]. It essentially consists of treating the active enzyme precipitate in situ with a crosslinking reagent (mostly glutaraldehyde is used). Fig. 2 describes what happened when the glutaraldehyde concentration was varied while preparing the CLEA of a lipase [44]. Higher crosslinking reagent concentration makes the protein more rigid. This could be correlated with high half-lives (at 55 °C), lower hydrolytic activity, and more drastic decrease in initial rates of transacetylation in solvent free medium. What was most interesting was that enantioselectivity (E) improved considerably upon mild crosslinking and dropped when more crosslinks were introduced. So, there is an optimum for protein rigidity for obtaining best enantioselectivity. More is not always better. It is obvious that optimum rigidity will be different for each enzyme and will depend upon the reaction parameters like temperature, nature of the reaction medium and *a*_w_ of the reaction medium for any enzyme [56].

In organic solvents, higher *a*_w_ and temperature, both increase the flexibility of the enzyme molecule. Many years back, Mattiasson’s group, in a series of papers, had studied the effect of varying *a*_w_ and temperature on the enantioselectivity of alcohol dehydrogenase (ADH) from *Thermoanaerobacter brockii* in the reduction of few ketones [75], [34], [35]. This reaction is a valuable way of obtaining chiral alcohols. Some interesting observations were as follows: the enantioselectivity was different for different substrates both in aqueous medium as well as in hexane [75]. With 2-pentanone as a substrate in hexane, low temperatures favoured enantioselectivity (with S-alcohol as the major product). Expectedly, rates were lower at low temperature. With 2-butanone, enantioselectivity increased with increasing temperature but it was reversed as well (R-alcohol was the main product). The authors rationalized the result by pointing out that with both substrates, increase in temperature favoured the formation of R-alcohols. Surprisingly, increasing *a*_w_ increased the enantioselectivity [34]. The correlation between flexibility and enantioselectivity seems to be less than completely understood. More extensive discussion on this can be found at several places [56], [21].

In the case of hydrolases at least, the picture has become clearer over the years. We have referred to the effect of the treatment of subtilisin with 6 M urea and performance of the treated enzyme in *n*-hexane. Fig. 1 shows the effect of temperature at which the transesterification was carried out in hexane by dried subtilisin and urea-treated subtilisin [52]. At lower temperature when the protein flexibility decreased, the urea treated enzyme had 90× higher initial rates (as compared to the untreated enzyme) at 15 °C. Only at 10 °C higher reaction temperature (25 °C) this dropped to 50-fold. Interesting enough, adding small amount of water to the reaction medium (which is known to increase protein flexibility) had the similar effect. The urea treated enzyme showed lower enantioselectivity in the kinetic resolution of the unnatural substrate (*R*,*S*)-1-phenylethanol but higher enantioselectivity in the kinetic resolution of the natural substrate *N*-acetyl-(*R*,*S*)-phenylalanine ethyl ester [52]. This is in agreement with the excellent reasoning by Broos [8] who explains the interplay between flexibility and enantioselectivity for natural and unnatural substrates in terms of transition state theory.

Higher flexibility also seems to favour better catalytic activity for the promiscuous reactions. Promiscuous reactions are reactions wherein an enzyme catalyses a reaction type which is not in line with how it is classified under EC nomenclature system [37], [4], [45], [43]. Urea treated subtilisin again showed higher reaction rates for the aldol condensation between *p*-nitrobenzaldehyde and acetone in organic solvents [52].

## 6 Conclusion

The importance of conformational flexibility has been understood for a long time. The ΔΔ*G* for the N ↔ D transition in proteins is merely in the range of 5–20 kcal/mol [68] and arises out of the balance between enthalpy and entropy terms in Δ*G* = Δ*H *− *T*Δ*S* equation. It is just that the two developments in recent decades have created a need for us to look at them with renewed interest more closely. First is the possibility of carrying out reactions in a wide range of non-aqueous media. We have recently pointed out that the structures of enzymes do not become rigid in nearly anhydrous organic solvents (as is often implied) [52]. These become rigid at the drying stage and do not get a chance to acquire the necessary flexibility in such solvents unless water or other H-bonding solvents like DMF/DMSO are added. Hence, how we dry enzymes prior to placing these in such media is important. Benefits of the presence of lyoprotectants and cryoprotectants during lyophilization has been known for some time [58], [2], [60]. It seems that drying by precipitating with organic solvents may be better [59], [62], [63], [66], [42]. What is interesting is that at least in the case of alpha chymotrypsin and subtilisin, its makes a difference whether organic solvent is added to the aqueous solution of the enzyme or vice versa [67].

The sub context is that we need to pursue more vigorously the role which flexibility plays for defining enantioselectivity and in relatively more recently discussed catalytically promiscuous reactions.

The second development is more recent and it is a little early to understand its importance completely. Intrinsically disordered proteins (IDPs) challenge our entrenched thinking in terms of structure–function paradigm. In these cases, the total flexibility in large parts of the protein molecules define the biological role. The “hub” proteins recognize and bind to many different but a specific set of ligands [49]. The molecular recognition presumably operates via induced fit mechanism. Let us step back a little and refer to the old and now buried debate on “selection” versus “instruction” theories on generation of antibodies [9]. The proponents of selection theories turned out to be correct and clonal selection theory is a part of the standard texts on immunology or even biochemistry [31], [6]. Many current approaches in biotechnology are inspired by this philosophy. The directed evolution technology [3] for tailoring biocatalyst designs, peptide libraries and combinatorial approach, all rely upon selecting the right candidate from a large pool [39]. IDPs seem to tell us that there may be possibilities of developing another set of strategies inspired by “instruction” school of thought led by Pauling. A given protein moulds itself to become its receptor. In a way, molecular bioimprinting of proteins shows that it is possible. Perhaps, that is just scratching the surface. It seems that we still have not heard the last word on the importance of flexibility in biocatalysis.

## References

[1] Adlercreutz P. (2013). Immobilisation and application of lipases in organic media. Chem. Soc. Rev..

[2] Arakawa T., Prestelski S.J., Kenney W.C., Carpenter J.F. (2001). Factors affecting short-term and long-term stabilities of proteins. Adv. Drug Deliv. Rev..

[3] Arnold F.H., Georgiou G. (2003). Directed Evolution Screening and Selection Methods.

[4] Arora B., Mukherjee J., Gupta M.N. (2014). Enzyme promiscuity: using the dark side of enzyme specificity in white biotechnology. Sustain. Chem. Process..

[5] Bemporad F., Chiti F. (2009). Native-like aggregation of the acylphosphatase from Sulfolobus solfataricus and its biological implications. FEBS Lett..

[6] Berg J.M., Tymockzo J.L., Stryer L. (2002). Biochemistry.

[7] Brock T.D. (1986). Thermophiles, General Molecular and Applied Microbiology.

[8] Broos J. (2002). Impact of the enzyme flexibility on the enzyme enantioselectivity in organic media towards specific and non-specific substrates. Biocatal. Biotransform..

[9] Campbell D.H., Rich A., Davidson N. (1968). Antibody formation: from Ehrlich to Pauling and return. Structural Chemistry and Molecular Biology.

[10] Cao L. (2005). Carrier-bound Immobilized Enzymes: Principles Applications and Design.

[11] Carrea G., Riva S. (2000). Properties and synthetic applications of enzymes in organic solvents. Angew. Chem. Int. Ed..

[12] Cavicchioli R., Siddiqui K.S., Andrews D., Sowers K.R. (2002). Low temperature extremophiles and their applications. Curr. Opin. Biotechnol..

[13] De Diego T., Manjon A., Lozano P., Iborra J.L. (2011). A recyclable enzymatic biodiesel production process in ionic liquids. Bioresour. Technol..

[14] de Miguel Bouzas T., Barros-Velázquez J., Villa T.G. (2006). Industrial applications of hyperthermophilic enzymes: a review. Protein Pept. Lett..

[15] Engasser J., Horvath C., Wingard L.B., Katchalski-Katzir E., Golstein L. (1976). Diffusion and kinetics with immobilized enzymes.

[16] Fersht A. (1984). Enzyme Structure and Mechanism.

[17] Fessner W.D., Anthonsen T. (2008). Modern Biocatalysis: Stereoselective and Environmentally Friendly Reactions.

[18] Georlette D., Bentahir M., Claverie P., Collins T., D'Amico S., Delille D., Feller G., Gratia E., Hoyoux A., Lonhienne T., Meuwis M.-A., Zecchinon L., Gerday C.H., De Cuyper M., Bulte J.W.M. (2001). Cold Adapted enzymes. Physics and Chemistry Basis of Biotechnology.

[19] Gerday C., Aittaleb M., Arpigny J.L., Baise E., Chessa J.P., Garsoux G., Petrescu I., Feller G. (1997). Psychrophilic enzymes: a thermodynamic challenge. Biochim. Biophys. Acta.

[20] Gomes J., Steiner W. (2004). The biocatalytic potential of extremophiles and extremozymes. Food Technol. Biotechnol..

[21] Gotor V., Alfonso I., Garcia-Urdiales E. (2008). Asymmetric Organic Synthesis with Enzymes.

[22] Guisan J.M. (2013). Methods in Biotechnology: Immobilization of Enzymes and Cells.

[23] Guo Y., Clark D.S. (2001). Activation of enzymes for nonaqueous biocatalysis by denaturing concentrations of urea. BBA-Protein Struct. Mol. Enzymol..

[24] Gupta M.N. (1992). Enzyme function in organic solvents. Eur. J. Biochem..

[25] Gupta M.N. (2000). Methods in Non-aqueous Enzymology.

[26] Gupta M.N., Crosslinking techniques: applications to enzyme and protein stabilization and bioconjugate preparation, in: Himmel M.E., and Gergiou G., (Eds.), Biocatalyst design for stability and specificity, ACS Symposium series 516, Washington, 1993.

[27] Halling P.J. (2000). Biocatalysis in low-water media: understanding effects of reaction conditions. Curr. Opin. Chem. Biol..

[28] Hanefeld U., Gardossi L., Magner E. (2009). Understanding enzyme immobilization. Chem. Soc. Rev..

[29] Hayashi R. (2002). Trends in High Pressure Bioscience and Biotechnology.

[30] Hecht K., Wrba A., Jaenicke R. (1989). Catalytic properties of thermophilic lactate dehydrogenase and halophilic malate dehydrogenase at high temperature and low water activity. Eur. J. Biochem..

[31] Hood L.E., Weissman I.L., Wood W.B., Wilson J.H. (1984). Immunology.

[32] Hudson E.P., Eppler R.K., Clark D.S. (2005). Biocatalysis in semi-aqueous and nearly anhydrous conditions. Curr. Opin. Biotechnol..

[33] R. Jaenicke, Structure function relationship of hyperthermophilic enzymes, in: Himmel M.E., and Georgiou G., (Eds.), Biocatalyst design for stability and specificity, ACS Symposium series 516, Washington, 1993, pp. 56.

[34] Jonsson A., van Breukelen W., Wehtje E., Adlercreutz P., Mattiasson B. (1998). The influence of water activity on the enantioselectivity in the enzyme-catalysed reduction of 2-pentanone. J. Mol. Catal. B: Enzym..

[35] Jonsson A., Wehtje E., Adlercreutz P., Mattiasson B. (1999). Thermodynamic and kinetic aspects on water vs. organic solvent as reaction media in the enzyme catalysed reduction of ketones. Biochim. Biophys. Acta.

[36] Kendrew J.C., Rich A., Davidson N. (1968). Information and conformation in Biology. Structural Chemistry and Molecular Biology.

[37] Khersonsky O., Tawfik D.S. (2010). Enzyme promiscuity: a mechanistic and evolutionary perspective. Annu Rev. Biochem..

[38] Koshland D.E. (1994). The key–lock theory and the induced fit theory. Angew. Chem. Int. Ed. Engl..

[39] Labrou N.E. (2014). Protein Downstream Processing, Design, Development and Application of High and Low Resolution Methods.

[40] Lee Y.M., Dordick J.S. (2002). Enzyme activation for nonaqueous media. Curr. Opin. Biotechnol..

[41] Luheshi L.M., Dobson C.M. (2009). Bridging the gap: from protein misfolding to protein misfolding diseases. FEBS Lett..

[42] Majumder A.B., Gupta M.N. (2011). Increasing catalytic efficiency of *Candida rugosa* lipase for the synthesis of *tert-alkyl* butyrates in low water media. Biocatal. Biotrasform..

[43] Majumder A.B., Gupta M.N. (2014). Lipase-catalyzed condensationreaction of 4-nitrobenzaldehyde with acetyl acetone in aqueous–organic cosolvent mixtures and in nearly anhydrous media. Synth. Commun..

[44] Majumder A.B., Mondal K., Singh T.P., Gupta M.N. (2008). Designing crosslinked lipase aggregates for optimum performance as biocatalysts. Biocatal. Biotransform..

[45] Majumder A.B., Ramesh N.G., Gupta M.N. (2009). Lipase catalyzed condensation reaction with a tricyclic diketone-yet another example of biocatalytic promiscuity. Tetrahedron Lett..

[46] Mattiasson B., Adlercreutz P. (1991). Tailoring the microenvironment of enzymes in water-poor systems. Trends Biotechnol..

[47] Mingarro I., Abad C., Braco L. (1995). Interfacial activation-based molecular bioimprinting of lipolytic enzymes. Proc. Natl. Acad. Sci. U. S. A..

[48] Minteer S.M. (2011). Enzyme Stabilization and Immobilization: Methods and Protocols.

[49] Mittag T., Kay L.E., Forman-Kay J.D. (2010). Protein dynamics and conformational disorder in molecular recognition. J. Mol. Recognit..

[50] Morita R.Y. (1975). Psychrophilic bacteria. Bacteriol. Rev..

[51] Mozhaev V.V., Melik-Nubarov N.S., Sergeeva M.V., Siksnis V.A., Martinek K. (1990). Strategy for stabilizing enzymes part one: increasing stability of enzymes via their multi-point interaction with a support. Biocatal. Biotransform..

[52] Mukherjee J., Mishra P., Gupta M.N. (2015). Urea treated subtilisin as a biocatalyst for transformations in organic solvents. Tetrahedron Lett..

[53] Noll K.M. (2013). Thermophilic bacteria. Breners Encycl. Genet..

[54] Nosoh Y., Sekiguchi T., Gupta M.N. (1993). Protein Engineering for Thermostabilization.

[55] Orlich B., Schomäcker R. (2002). Enzyme catalysis in reverse micelles. Adv. Biochem. Eng. Biotechnol..

[56] Patel R.N. (2000). Stereoselective Biocatalysis.

[57] Richards F.M. (1992). Linderstrom-Lang and the Carlsberg laboratory: the view of a postdoctoral fellow in 1954. Prot. Sci..

[58] Roy I., Gupta M.N. (2004). Freeze-drying of proteins: some emerging concerns. Biotechnol. Appl. Biochem..

[59] Roy I., Gupta M.N. (2004). Preparation of highly active alpha-chymotrypsin for catalysis in organic media. Bioorg. Med. Chem. Lett..

[60] Roy, Sharma A., Gupta M.N. (2004). Obtaining higher transesterification rates with subtilisin Carlsberg in nonaqueous media. Bioorg. Med. Chem. Lett..

[61] Schoevaart R., Wolbers M.W., Golubovic M., Ottens M., Kieboom A.P., van Rantwijk F., van der Wielen L.A., Sheldon R.A. (2004). Preparation, optimization, and structures of cross-linked enzyme aggregates (CLEAs). Biotechnol. Bioeng..

[62] Shah S., Gupta M.N. (2007). Kinetic resolution of (+/−)-1-phenylethanol in [Bmim][PF6] using high activity preparations of lipases. Bioorg. Med. Chem. Lett..

[63] Shah S., Gupta M.N. (2007). Obtaining high transesterification activity for subtilisin in ionic liquids. Biochim. Biophys. Acta.

[64] Shah S., Sharma A., Gupta M.N. (2006). Preparation of crosslinked enzyme aggregates by using bovine serum albumin as a proteic feeder. Anal. Biochem..

[65] Sheldon R.A., Schoevaart R., Van Langen L.M. (2005). Crosslinked enzyme aggregates (CLEAs): a novel and versatile method for enzyme immobilization (a review). Biocatal. Biotransform..

[66] Solanki K., Gupta M.N. (2008). Optimizing biocatalyst design for obtaining high transesterification activity by a-chymotrypsin in non-aqueous media. Chem. Cent. J..

[67] Solanki K., Gupta M.N., Halling P.J. (2012). Examining structure-activity correlations of some high activity enzyme preparations for low water media. Bioresour. Technol..

[68] Sowdhamini P., Balaram P., Gupta M.N. (1993). Protein structure and stability. Thermostability of Enzymes.

[69] Straathof A.J.J., Adlercreutz P. (2005). Applied Biocatalysis.

[70] Torres-Salas P., del Monte-Martinez A., Cutino-Avila B., Rodriguez-Colinas B., Alcalde M., Ballesteros A.O., Plou F.J. (2011). Immobilized biocatalysts: novel approaches and tools for binding enzymes to supports. Adv. Mater..

[71] Turner P., Mamo G., Karlsson E.N. (2007). Potential and utilization of thermophiles and thermostable enzymes in biorefining. Microb. Cell Fact..

[72] Tyagi R., Gupta M.N. (1998). Chemical modification and chemical cross-linking for protein/enzyme stabilization. Biochemistry (Mosc.).

[73] Uversky V.N. (2011). Intrinsically disordered proteins from A to Z. Int. J. Biochem. Cell Biotechnol..

[74] Uversky V.N., Fink A.L. (2007). Protein misfolding, aggregation and conformational diseases. Molecular Mechanisms and Conformational Diseases, Part B.

[75] Yang H., Jonsson A., Wehtje E., Adlercreutz P., Mattiasson B. (1997). The enantiomeric purity of alcohols formed by enzymatic reduction of ketones can be improved by optimisation of the temperature and by using a high co-substrate concentration. Biochim. Biophys. Acta.

[76] Zaks A., Klibanov A.M. (1984). Enzymatic catalysis in organic media at 100 degrees C. Science.

